# Factors affecting the *in vitro* embryo production in buffalo (*Bubalus bubalis*): A review

**DOI:** 10.17221/48/2022-VETMED

**Published:** 2023-02-21

**Authors:** Satish Kumar, Maiana Silva Chaves, Ana Flavia Bezerra da Silva, William Gomes Vale, Sebastiao Tavares Rolim Filho, Jose Carlos Ferreira-Silva, Luciana Magalhaes Melo, Vicente Jose de Figueiredo Freitas

**Affiliations:** ^1^Laboratory of Physiology and Control of Reproduction, State University of Ceará, Fortaleza, Brazil; ^2^Postgraduate Program in Veterinary Science, State University of Ceará, Fortaleza, Brazil; ^3^Animal Reproduction Sector, Federal Rural University of the Amazon, Belém, Brazil; ^4^Animal Reproduction Sector, Federal Rural University of Pernambuco, Recife, Brazil; ^5^Molecular Genetics Research Unit, University Center Fametro, Fortaleza, Brazil

**Keywords:** calving rate, embryo transfer, IVEP, oocytes, OPU, pregnancy rate

## Abstract

Under natural and well-managed conditions, the buffalo has good reproductive and productive indices. However, *in vitro* embryo production (IVEP) has been used commercially to maximise the number of elite animals. In this species, several factors (donor management, *in vitro* culture medium, semen, *in vitro* conditions, embryo transfer) still affect the IVEP results. In addition, the cost of this technique is very high for this purpose. Therefore, more studies, as well as adequate plans, are needed to achieve this objective efficiently. In this review, we discussed the current commercial status, influencing factors (*in vivo* and *in vitro*), and the progress and future challenges of IVEP in buffalo. A total of 81 references were used from 1979 to 2022. The relevant data or literature were searched using the following databases: Google, ResearchGate, Science Alert, Science Direct and PubMed, using the following keywords: buffalo oocytes/COCs, buffalo embryos, pregnancy and calving or live birth rate after embryo transfer. The best maturation, cleavage and blastocyst rates in the *in vitro* production of buffalo embryos were 95.8, 75.2 and 33.4%, respectively. The pregnancy and live birth rates ranged from 22.2% to 43.5% and from 15.3% to 36.5%, respectively, after the transfer of fresh embryos produced *in vitro* to the recipients. This review will help to contextualise IVEP in buffaloes, as well as create an adequate plan for implementing IVEP in buffaloes.

## INTRODUCTION

Buffaloes are considered suitable animals for the production of milk, meat and work (de la Cruz-Cruz et al. 2014). They are reared in different parts of the world, whose social and economic importance has been recognised in recent years. The productivity of buffalo is limited by inherent reproductive characteristics. It is a short day polyoestrus species with an average length of the oestrus cycle and oestrus duration of 21 days and 24 h, respectively (Harun-Or-Rashid et al. 2019). However, in tropical areas near the equator, they breed throughout the year. Independently of the area, the intensity of the oestrus expression is lower than cattle (Roy and Prakash 2009). The numbers of primordial follicles in buffalo (approximately 12 000–19 000) (Samad and Nasseri 1979) are lower than cattle (approximately 109 673 ± 86 078) (Silva-Santos et al. 2011). The buffalo typically shows two follicular waves (63.3%) (Jan et al. 2020) with a lower number of follicles recruited per follicular wave (Campanile et al. 2010) during an oestrus cycle than cattle.

To overcome these physiological problems, assisted reproductive technologies (ART), such as timed-artificial insemination (TAI), superstimulation, ovum pick-up (OPU), *in vitro* embryo production (IVEP) and embryo transfer (ET) have been introduced to increase the number of offspring of genetically elite buffaloes. However, the combined use of OPU and IVEP have been used to obtain promising results after the low efficiency and limited commercial application of *in vivo* embryo production in buffaloes (Baruselli et al. 2020). However, the buffalo’s breeding efficiency is excellent and depends on the management conditions. The first service conception rate (in optimum management conditions) in the buffalo is 65% and 40–50% with natural mating and cryopreserved semen, respectively. In well-organised management, the pregnancy rates can be up to 80% yearly with 14 to 15 months calving intervals (Perera 2008).

In this review, we discussed the current commercial status, influencing factors (*in vivo* and *in vitro*), and the progress and future challenges of IVEP in buffaloes. A total of 81 references were used from 1979 to 2022.

The relevant data or literature were searched using the following databases: Google, ResearchGate, Science Alert, Science Direct and PubMed, using the following keywords: buffalo oocytes/COCs, buffalo embryos, pregnancy and calving or live birth rate after embryo transfer.

## CURRENT COMMERCIAL SITUATION OF IVEP IN BUFFALOES

All the studies (Table 1) proved that IVEP in buffaloes is at a commercial level, and the results are continuously improving. However, the cost of IVEP is 3 to 4 times higher than zebu cattle (Ohashi et al. 2017). Therefore, it is necessary to understand the responsible factors that affect the outcome of this technique for further improvement.

**Table 1:** Pregnancy and birth rates after the transfer of fresh embryos in oestrus synchronised buffalo re-cipients

Pregnancy rate % (*n*)	Birth rate % (*n*)	References
41.3 (12/29)	26.9 (7/26)*	Liang et al. (2007)
34.4 (10/29)	15.3 (4/26)	
43.5 (50/115)	36.5 (42/115)	Saliba et al. (2013)
24.0 (6/25)	–	Ohashi et al. (2017)
22.2 (19/89)	–	Marin et al. (2019a)
43.0 (49/114)	35.1 (40/114)	Saliba et al. (2020)

*Natural oestrus

## FACTORS AFFECTING THE *IN VITRO* EMBRYO PRODUCTION IN BUFFALOES

### Donor

The selection of donors and their management is an important step for the OPU-IVEP technology. The selection directly affects the efficiency of this technology. The body condition score (BCS) and weight of the animals show the nutritional and management status of the farm. The nutrition influences the follicular dynamics, morphology, quality and developmental capacity of oocytes and *in vivo* and *in vitro* embryo development (Ohashi et al. 1998). It was observed that the mean aspiration rate of the oocytes per OPU per buffalo is 8.9 ± 5.0, with a range of 0 to 30 oocytes (Baruselli et al. 2018). Therefore, the selection of the donor is an important step and it can be performed by ultrasound or conducting an anti-Müllerian hormone (AMH) assay. The ovarian antral follicular populations (AFPs) are correlated positively with the plasma AMH concentrations in buffaloes (Liang et al. 2016). In turn, the AMH correlates positively with the superovulation response in buffaloes (Redhead 2017). It is worth noting that Kavya et al. (2017) did not observe any correlation of the serum AMH with the AFP and body weight (b.w.), but the AFP was correlated highly with the b.w. in Murrah buffalo heifers.

Baruselli et al. (2018) observed that the viability of the oocyte and blastocyst rates were not affected by the number of oocytes recovered per OPU. Though, the blastocyst rate was higher when the recovery rate of the oocytes per OPU was higher. In contrast, the pregnancy rate after embryo transfer (ET) in buffaloes was lower in the donors with a higher recovery rate of oocytes per OPU.

When evaluating some variables (Table 2), it was found that different categories influenced (*P* < 0.05) the overall process. In addition, the post-partum period and parity of the donors did not affect the IVEP efficiency in buffaloes (Baruselli et al. 2018).

**Table 2:** Effect of the management, health status and selection of the donor during the *in vitro* embryo production

Variables	Number of aspirated oocytes (mean ± SEM)	Blastocyst rate (%)	References
Parity			
Nulliparous	6.3	13.3	Gamarra et al. (2015)
Multiparous	11.1	18.3	
Age			
8–12 years	71.0^a^	–	Aquino and Atabay (2014)
13–17 years	29.0^b^	–	
Category			
Pre-pubertal calves	10.9 ± 3.3^ab^	5.1^b^	Silva et al. (2017)
Heifers	15.5 ± 2.1^a^	9.3^a^	
Lactating buffaloes	5.8 ± 1.3^b^	15.4^a^	

^a,b^Superscripts within a column differed significantly for each research at *P* < 0.05

### Seasons (breeding or non-breeding)

In buffaloes, the oocytes aspiration rates from slaughterhouse ovaries or the OPU (Di Francesco et al. 2012), *in vitro* oocyte maturation (IVM) and blastocyst rates were decreased during the non-breeding season (Sadeesh et al. 2016). Also, the pregnancy losses were higher in the non-breeding season during artificial insemination (Qayyum et al. 2018) or after the transfer of *in vitro* produced embryos in buffaloes (Saliba et al. 2020). These results might be due to the increased daylight length with higher environmental temperature leading to hyperprolactinemia, the decreased secretion of gonadotropins and steroids. These factors affect the folliculogenesis, oocyte quality and embryonic development (Diaz et al. 2020). The degeneration rate of the cumulus cells and ooplasm are also higher in the hot than in the cold season without affecting the expansion of the cumulus cells during *in vitro* maturation. In addition, the *in vitro*-produced embryo or preimplantation embryo quality decreases during heat stress. The glucose metabolic genes and apoptotic or developmental competent genes are downregulated and upregulated, respectively. The number of essential molecules needed for early embryonic development is impaired and causes chromosomal aberration (Sadeesh et al. 2016). Biological functions, such as mitochondrial transcription, replication, apoptosis and the production of chaperones are altered during the hot season (Ferreira et al. 2013). Therefore, IVEP should be conducted in the breeding season.

### Oocytes

The number of oocytes and their quality directly affect the blastocyst rates. These are affected by the follicular size, the source of the oocytes, the ovary transportation conditions and recovery methods.

With regard to aspects related to the transportation of ovaries obtained from slaughterhouses, the temperature should be maintained at 25 °C to 30 °C and the oocytes should be aspirated and processed within 6 h of slaughter (Di Francesco et al. 2007). Buffalo oocytes are prone to cellular damages due to autolytic processes, especially when the excised ovaries are placed outside for a long period (Neglia et al. 2003). Also, buffalo oocytes are more sensitive to oxidative damage due to the high lipid content in the ooplasm (Marin et al. 2019b).

The cumulus oocyte complexes (COCs) can be obtained through aspiration, scoring and slicing methods for IVEP. In buffaloes, studies have shown that these methods can affect the quantity and quality of the oocytes. The aspiration method gives better (*P* < 0.05) results (IVM and blastocyst rates) than the scoring and slicing methods for IVEP (Habeeb et al. 2019). However, the number of retrieved oocytes was more for these methods than the aspiration method. The lower development competence of the oocytes obtained from the scoring or slicing methods may be due to them being embedded deep in the cortex of the ovary (Arlotto et al. 1990). In addition, it was also observed that the oocytes obtained from deeper situated follicles in the cortex of the ovary have lower meiotically development competence than surface-situated follicles in bovines (Arlotto et al. 1990).

Studies revealed that the COCs with more layers of *cumulus* cells with a homogenous ooplasm (Waheed et al. 2016) retrieved from large follicles (> 8 mm) (Raghu et al. 2002) had comparatively higher developmental competence than fewer *cumulus* cells and smaller follicles in buffaloes. The *cumulus* cells help in the nuclear and cytoplasmic maturation by transducing the luteinising hormone (LH) signal to the oocyte (Medeiros et al. 2021). They protect the oocytes against oxidative stresses. They locally produce glycosaminoglycans, steroid hormones and other factors (Turathum et al. 2021). Also, they help transport nutrients and signals in or out of the oocytes. Regarding the follicular size, the expression of the competence marker genes, such as *GDF9* and *BMP15* in the oocyte; *GREM1*, *PTGS2*, *HAS2* and *EGFR* in the *cumulus* cells are upregulated in the COCs retrieved from ≥ 6 mm follicles (Pandey et al. 2018). Furthermore, COCs derived from larger sized follicles have more cytoplasmic content, responsible for their cytoplasmic maturation and further development (Raghu et al. 2002). During the aspiration of the oocytes, a greater number of grade-A oocytes were obtained from the slaughterhouse (SH) sourced ovaries than the OPU in buffaloes (Neglia et al. 2003). It may be due to the more mechanical damage of the granulosa cells during OPU and is responsible for the improper evaluation of the oocyte quality during OPU. In contrast, the blastocyst rate was higher for oocytes collected from live buffaloes by the OPU method than from SH ovaries (Neglia et al. 2003). Therefore, it is advisable to select good quality oocytes, use the aspiration method of oocytes collected from slaughterhouse ovaries and the oocytes should be processed as soon as possible after aspiration during IVEP.

## HORMONAL TREATMENT, ASPIRATION FREQUENCY AND OVARIAN STATUS DURING OPU

The hormone treatment before OPU improved the results during the IVEP programme. The proportion of viable oocytes for culture and efficiency of IVEP were enhanced by superstimulation with the follicle-stimulating hormone (FSH) (de Carvalho et al. 2019) and in combination with the gonadotropin-releasing hormone (GnRH) (Sakaguchi et al. 2019) before the OPU in buffalo donors. It is believed that the FSH prevents the disruption of the cell-to-cell connection (the connection between the oocytes, *cumulus* cells, and each *cumulus* cell) (Sugimura et al. 2017). It was also reported that the FSH increased the proportion of medium-sized follicles and made them available for the OPU (Baruselli et al. 2018). In addition, studies observed that the bovine somatotropin (bST) treatment increases the number of antral follicles recruited per follicular wave in buffaloes (Sa Filho et al. 2009; Ferraz et al. 2015). The mechanism of the effect of bST on the follicular wave is not clearly understood. It is believed that it increases the concentration of insulin-like growth factor-I (IGF-I) and insulin in the circulation, increasing the number of antral follicles (Bilby et al. 2006).

As for the frequency of the follicular aspiration, there is not a fixed protocol available for the aspiration frequency of oocytes in buffaloes (Table 3). In bovine, the oocyte recovery, quality, cleavage and blastocyst rate are influenced by the phases of the oestrus cycle (Vassena et al. 2003). However, in buffaloes, the OPU-IVEP efficiency was not affected by the OPU performed on days 1, 3 and 5 of the follicular wave emergences (Gimenes et al. 2015). It has also been observed that the number of available follicles was reduced for aspiration during the luteal phase in buffaloes (Deb et al. 2020). It may be due to the lutein cells of the *corpus luteum* (CL) occupying a portion of the ovary that inhibits the follicular development and promotes atresia (Hafez 1993; Deb et al. 2020). Besides, the progesterone secreted by the lutein cells impedes the follicular development and increases the atresia (Hafez 1993).

**Table 3:** Effect of the aspiration frequency on the *in vitro* embryo production

Aspiration frequency	Recovered oocytes (%)	Cleavage rate (%)	Blastocyst rate (%)	Blastocyst/buffalo/session (mean ± SEM)	References
Twice a week (control)	57.7	41.7	26.0	1.2 ± 0.2	Sa Filho et al. (2009)
Twice a week (bST)	54.5	46.2	19.7	1.3 ± 0.6	
14-day interval	73.6^a^	32.7	19.5	1.7 ± 0.4	Ferraz et al. (2015)
7-day interval	69.3^a^	33.4	18.6	1.3 ± 0.2	
14-day interval + bST	58.5^b^	26.0	13.4	0.8 ± 0.2	
7-day interval + bST	67.4^a^	35.1	9.6	0.7 ± 0.1	
14-day interval	51.0^a^	64.0^a^	28.0^a^	–	Konrad et al. (2017)
7-day interval	31.5^b^	44.0^a^	6.0^b^	–	
7-day interval	76.0	–	23.0	–	Marin et al. (2019a)

^a,b^Superscripts within a column differed significantly for each research at *P* < 0.05

bST = bovine somatotropin

## *IN VITRO* CULTURE CONDITIONS

### Different concentration of O_2_ during IVEP

The oocyte maturation and blastocyst rates were affected by the different concentrations of O_2_ used during IVEP in buffaloes. Studies revealed that 5% O_2_ improves the *in vitro* oocyte developmental competence as compared to 20% in domestic animals (Leite et al. 2017) including buffaloes (Kumar et al. 2015). It is justified that the *in vitro* oxygen concentration mimics the *in vivo* oxygen tension in the oviduct of mammals, which can vary between 2% to 8% and drops to 2% in the uterine milieu (Sciorio and Smith 2019).

In addition, embryos produced *in vitro* under 20% O_2_ in ruminant animals differ in the metabolism of *in vivo* embryos including higher aerobic glycolysis, greater lactate production, and higher lactate oxidation (Khurana and Niemann 2000). Moreover, 20% O_2_ concentration during *in vitro* culture increases the level of harmful reactive oxygen species within the cells and reduces the embryo development to the blastocyst stage by changes to the transcriptome, proteome, metabolic gene expression, epigenome (Leite et al. 2017) and inducing premature X-chromosome inactivation (Lengner et al. 2010).

### Adding supplements to the media used in IVEP

During *in vivo* conditions, the secretion of follicular and oviductal fluids provide sufficient antioxidants and other supplements to the embryo for its development. However, during *in vitro* culture, it depends upon the *in vitro* culture medium conditions. The studies revealed that different supplements to the IVM or *in vitro* embryo culture (IVC) medium play an important role for the oocyte developmental competence during IVEP when used in an optimal concentration (Marin et al. 2019b). The culture system in this species is improving continuously. The prepubertal oocytes obtained from calves have lower developmental competence, and adults have more chromosomal aberration during *in vitro* conditions (Baldassarre 2021). In recent years, the blastocyst rate in buffaloes has increased (> 35%) due to improving the *in vitro* maturation and fertilisation conditions (Table 4). The studies observed that the *in vitro* culture of a synthetic oviductal fluid (SOF) medium is suitable for buffaloes as used in bovines (Pereira 2015; Pandey et al. 2018). Despite this, the pregnancy and calving rates are still low compared to the bovine.

**Table 4:** Effect of the different supplements (best results of the different concentrations grouped in a study) to IVM, IVF and IVC medium during *in vitro* embryo production in buffalo

Supplements*	Concentrations*	M	MR (%)	CR (%)	BR (%)	Functions	References
Glucose	20 mM	IVM	94.8	–	28.9	energy source	Kumar et al. (2015)
Essential oil of *Lippia origanoides*	2.5 μg/ml	IVM	78.1	45.9	35.05	antimicrobial and antioxidant	Pereira (2015)
MSCs-CM + mSOF	50% + 50%	IVC	–	70.1	24.2	secrete several cytokines or growth factors	Bhardwaj et al. (2016)
Melatonin	250 μM	IVM	69.7	–	–	antioxidant and anti-apoptotic	Nagina et al. (2016)
Heparin + caffeine	0.02 mg/ml + 3.89 mg/ml	IVF	–	56.2	22.2	sperm capacitating agents	Waheed et al. (2016)
α-linolenic acid	100 μM	IVM	76.3	57.8	21.0**	cytoplasmic maturation	Azam et al. (2017)
PHE	25 μl/ml	IVF	–	46.1	–	sperm capacitating agents	El-Ruby et al. (2017)
Ascorbic acid	50 μM	IVM	–	67.6	12.8	antioxidant	El-Naby et al. (2017)
Ascorbic acid + cysteamine	50 μM + 50 μM		–	65.2	12.3		
Oviduct- specific glycoprotein	10 μg/ml	IVF	–	75.2	25.0	paracrine regulator	Choudhary et al. (2017)
9-*cis*RA	5 nM	IVM	95.8	61.1	–	promotes maturation	Gad et al. (2018)
Roscovitine	50 μΜ	IVM	–	51.0	30.5	meiotic inhibitor and enhance developmental competence	Pandey et al. (2018)
Sericin	0.05%	IVM	89.2	–	–	antioxidant and improve nuclear maturation	Gustina et al. (2019)
FBS	10%	IVM	–	63.6	30.7	growth factors and prevents the hardening of the zona pellucida + antioxidant	Marin et al. (2019b)
FBS + L-carnitine	10% + 3.03 mM	IVC	–	60.2	17.6		
FBS + L-carnitine	10% + 3.03 mM	IVM-IVC	–	55.4	21.6		
BSA + L-carnitine	0.4% + 3.03 mM	IVM	–	53.4	33.4		
L-carnitine	1 mM	IVC	–	63.5	24.3	antioxidant	El-Sokary et al. (2021)

*Supplements and their best results from the concentrations used in the *in vitro* mediums during IVEP; **Morula mesenchymal stem cells conditioned medium (MSCs-CM)

BR = blastocyst rate; BSA = bovine serum albumin; CR = cleavage rate; FBS = fetal bovine serum; IVC = *in vitro* embryo culture; IVF = *in vitro* fertilisation; IVM = *in vitro* oocyte maturation; M = medium; MR = maturation rate; MSCs-CM = mesenchymal stem cells-conditioned medium; mSOF = modified synthetic oviductal fluid; PHE = D-penicillin, hypotaurine, and epinephrine

Buffalo oocytes and embryos are more susceptible to oxidative damage due to their high lipid content (Marin et al. 2019b). This oxidative damage can be overcome by using supplements to the medium with hypotaurine, taurine, enzymes (superoxide dismutase, glutathione peroxidase, and gamma glutamyl-cysteine synthetase), vitamins and antioxidants. The addition of the FSH to the IVM media increases the *in vitro* maturation rate (Marin et al. 2019a) and the mRNA expression of the FSH and LH receptors in the *cumulus* cells.

During the fertilisation process, biophysical, biochemical, molecular and metabolic changes occur. Some capacitating agents are secreted in the female reproductive tract during *in vivo* fertilisation (Maitan et al. 2022), but need to add these agents in the *in vitro* fertilisation (IVF) medium during *in vitro* conditions (Reckova et al. 2015). These agents, heparin, caffeine, calcium ionophore and PHE (D-penicillin, hypotaurine, and epinephrine) have been used singly or combined with others (Waheed et al. 2016; El-Ruby et al. 2017). The different supplements used (the best results of different concentrations grouped in a study) for the IVM, IVF and IVC medium during IVEP in buffaloes are presented in Table 4.

## SEMEN

The *in vitro* fertilisation is affected by the sperm quality and breeds (Longobardi et al. 2020). Different buffalo breeds affect the IVEP process (Soliman et al. 2018a), but different bulls (high reproductive performance) of the same breed do not affect the blastocyst rate (Marin et al. 2019a). Soliman et al. (2018b) observed that the IVF cleavage and blastocyst rate is affected by the different breeds of bulls in buffaloes. It may be due to the bull factors or the differences in the metabolic activity of the sperm cells (Longobardi et al. 2020).

The process of sperm sorting is related to several molecular changes, such as increased levels of reactive oxygen species (Tvrda et al. 2016), increased membrane permeability, and lower intracellular adenosine triphosphate (ATP) levels. These molecular changes decrease the sperm motility, viability, longevity and induce DNA fragmentation, and are responsible for the impaired membrane fusion and poor fertilisation rates (Neculai-Valeanu and Ariton 2021).

Limited studies are available for the use of sex-sorted semen during IVEP in buffaloes. Lu et al. (2007) observed a significantly higher cleavage (42 vs. 20) and blastocysts rate (52 vs. 29) for unsorted than sorted sperm, respectively.

In contrast, Liang et al. (2008) did not observe any significant differences between the cleavage (50.5 vs. 50.9), blastocysts (15.3 vs. 19.1) and pregnancy (5/43–11.6 vs. 7/26–26.9) rate when using sexed and unsexed semen, respectively. Also, Gamarra et al. (2015) did not observe any significant differences between the cleavage and blastocyst (20 vs. 17) rate when using sexed and unsexed semen, respectively.

The cryopreservation process decreases the activity of antioxidant enzymes, membrane integrity and increases the nuclear DNA fragmentation (Kumar et al. 2022). The IVF rate is affected by the cryopreserved semen used for IVF (Soliman et al. 2018a). Also, the live sperm percentage and motility of the spermatozoa drop by freezing up to 61.8% and 42.5%, respectively (Mahmoud et al. 2015). Soliman et al. (2018a) obtained a higher blastocyst rate when using fresh semen for IVF than cryopreserved semen during IVEP in buffaloes. Recently, Almeida et al. (2020) obtained better results during IVF in buffaloes while using chilled (for 24 h at 5 °C) semen than cryopreserved semen.

## ADVANCEMENTS AND FUTURE CHALLENGES

The IVEP technology in buffaloes started from the adaptation of the one used in cattle with some modifications. Nowadays, due to the growing market and genetic improvement of the buffalo herd, IVEP in this species already presents changes consistent with its physiology. Currently, it is known that low blastocyst rates are related to the low number of antral follicles available for OPU, management of buffaloes, and *in vitro* culture conditions. Many factors are responsible for the lower pregnancy or calving rates after the transfer of *in vitro* produced embryos. In bubaline, during an *in vitro* embryo production and transfer programme, chromosomal aberrations (Yoshizawa et al. 2010), insufficient progesterone concentration (Saliba et al. 2020) and improper contact of trophectoderm to the endometrium have been reported. In addition, errors in the elongation and attachment of the conceptus, germinal disc diameter, yolk sac development, binucleated cell numbers and foetal growth trajectory alteration have been reported in bovines (Ealy et al. 2019). Therefore, despite the progress of IVEP in buffaloes, more factors or techniques still need to be studied and understood to improve the outcome.

In this regard, Saliba et al. (2020) observed that synchronisation protocols and recipient *corpus luteum* diameter influence the pregnancy and parturition rates after an embryo transfer in buffaloes. They found that Ovsynch (ovarian synchronisation) was superior to progesterone combined with estradiol and an ECG-based protocol. They also observed that the pregnancy rate was better when recipients had a *corpus luteum* diameter greater than or equal to 14.5 mm. Some other specific points in developing a sequential culture system deserve attention, such as the embryo development in buffaloes is 12 h to 24 h faster than in bovines (Galli et al. 2001). Furthermore, the early stages of *in vitro* development (up to day 4) of buffalo embryos require high concentrations (1.5 mM) of glucose (Kumar et al. 2015). A major challenge is to develop a sequential culture system or an automated media change (AMC) system according to their developmental stages. An AMC system will also be helpful to avoid the stress of external incubator manipulations. Recently, Lakshmi Devi et al. (2022) used a uterine epithelial cell monolayer *in vitro* culture system supplemented with progesterone (3.14 ng/ml) and estradiol-17b (10 pg/ml). So, this type of cultural system also opens a new way of thinking.

New emerging technologies, i.e., transcriptomics, proteomics and metabolomics can help develop this system (Sugimura et al. 2017; Kumar et al. 2020). In addition, the Digital Embryo Development Monitoring Analysis and archiving system (PrimoVision) or Light Sheet fluorescent microscope can help to achieve this goal.

In buffaloes, prepubertal calves have also been used for the IVEP programme, but the results were not satisfactory (Baldassarre 2021). The main reason is the lack of knowledge about hormonal priming before the aspiration of oocytes in prepubertal calves and suitable *in vitro* maturation media for it. Another point, for commercial purposes, pregnancy rates can be improved if parthenotes are transferred with the *in vitro* fertilised embryos. The hypothesis is that parthenogenetic embryos secrete interferon-tau which will help in the implantation. It can also be used to deeply study the mechanism of the molecular communication between the embryo and the uterus during implantation.

## FINAL CONSIDERATIONS

In buffaloes, the use of *in vitro* embryo production is one of the tools that can be used to maximise their production. In this sense, studies have already shown that some factors affect the results and it is possible to minimise their impacts during IVEP, through the selection of donors by the AMH dosage, verifying the available follicles for OPU by ultrasound and applying the appropriate hormonal treatment before OPU, considering the season, ovarian transport time, follicular size, use of supplements in IVM, IVF and IVC, and the quality of semen used in the procedure.

The contextualization of the intrinsic aspects involved in the PIVE of buffaloes shows the tools that can improve the results obtained from this technique and help to improve the genetics of the buffalo herd.
